# Mental health in the slums of Dhaka - a geoepidemiological study

**DOI:** 10.1186/1471-2458-12-177

**Published:** 2012-03-09

**Authors:** Oliver Gruebner, M Mobarak H Khan, Sven Lautenbach, Daniel Müller, Alexander Krämer, Tobia Lakes, Patrick Hostert

**Affiliations:** 1 Geomatics Lab, Geography Department, Humboldt-Universität zu Berlin, Berlin, Germany; 2 Department of Public Health Medicine, School of Public Health, University of Bielefeld, Bielefeld, Germany; 3 Department for Computational Landscape Ecology, UFZ-Helmholtz Centre for Environmental Research, Leipzig, Germany; 4 Leibniz Institute of Agricultural Development in Central and Eastern Europe (IAMO), Halle (Saale), Germany; 5 Department of Urban Planing and Real Estate Management, Institute of Geodesy and Geoinformation- IGG Bonn, Universität Bonn, Bonn, Germany

## Abstract

**Background:**

Urban health is of global concern because the majority of the world's population lives in urban areas. Although mental health problems (e.g. depression) in developing countries are highly prevalent, such issues are not yet adequately addressed in the rapidly urbanising megacities of these countries, where a growing number of residents live in slums. Little is known about the spectrum of mental well-being in urban slums and only poor knowledge exists on health promotive socio-physical environments in these areas. Using a geo-epidemiological approach, the present study identified factors that contribute to the mental well-being in the slums of Dhaka, which currently accommodates an estimated population of more than 14 million, including 3.4 million slum dwellers.

**Methods:**

The baseline data of a cohort study conducted in early 2009 in nine slums of Dhaka were used. Data were collected from 1,938 adults (≥ 15 years). All respondents were geographically marked based on their households using global positioning systems (GPS). Very high-resolution land cover information was processed in a Geographic Information System (GIS) to obtain additional exposure information. We used a factor analysis to reduce the socio-physical explanatory variables to a fewer set of uncorrelated linear combinations of variables. We then regressed these factors on the WHO-5 Well-being Index that was used as a proxy for self-rated mental well-being.

**Results:**

Mental well-being was significantly associated with various factors such as selected features of the natural environment, flood risk, sanitation, housing quality, sufficiency and durability. We further identified associations with population density, job satisfaction, and income generation while controlling for individual factors such as age, gender, and diseases.

**Conclusions:**

Factors determining mental well-being were related to the socio-physical environment and individual level characteristics. Given that mental well-being is associated with physiological well-being, our study may provide crucial information for developing better health care and disease prevention programmes in slums of Dhaka and other comparable settings.

## Background

Mental health conditions are of rising concern as they increasingly contribute to the global burden of disease [1]. Neuropsychiatric disorders (including depression, alcohol-and substance abuse, or psychoses) add to the so-called disability-adjusted life-years (DALYs). This contribution is further projected to increase worldwide, from 13.5% in 2005 to 14.4% in 2030 [2,3].

In low-income countries for example, depression has become almost as prevalent as malaria (3.2% versus 4% of the total disease burden) [4] and this number is projected to further increase to ~5% in 2030 [2]. However, mental health issues tend to be overtaken by other health problems, especially in the rapidly urbanising megacities of developing countries, where a growing number of people are living in slums and unhealthy environments [5]. High levels of environmental pollution, lack of adequate water and sanitation, overcrowding, insecurity of tenure, and non-durability of housing could adversely affect the health of slum dwellers [6-8]. To date, little is known about the burden of disease in urban slums [9] and research on the mental well-being of slum residents is lacking [10].

Dhaka, the capital of Bangladesh, is one of the fastest growing megacities in the world and in 2005, approximately 3.4 million out of the city's 12.6 million inhabitants were living in slums [11,12]. Today, the city comprises approximately 14 million inhabitants [12] with more than 300,000 new migrants, mainly the rural poor, moving to Dhaka each year [13,14]. As most of these new immigrants initially concentrate in slums [15,16], Dhaka's population growth led to an increase in the proportion of slum dwellers from 20% in 1996 to 37% in 2005 [11], which presents a daunting challenge for local health authorities [11,17].

The lack of data on the burden of disease morbidity and mental health status in slums hampers the efficient allocation of health care initiatives and the provision of appropriate disease prevention services [9]. Given that psychological well-being is associated with physiological well-being [3], assessing the factors that describe the mental well-being of poor populations residing in urban slums is urgently needed.

In this paper, we applied a geo-epidemiological approach combining very high-resolution land cover information with geo-referenced survey data for obtaining exposure information i.e., both pathogenetic and salutogenetic factors from the socio-ecological environment and personal characteristics. We assumed that these factors contribute to the mental well-being among slum residents in Dhaka. A socio-ecological conceptualisation of the environment allows for identifying both health threatening and health-promoting physical and social features of environments. As such these features are assumed to affect the emotional, physical, and social well-being of individuals and groups [cf. [18]]. Since mental well-being and physical well-being are interrelated [3], we used the findings of Gruebner et al. [19] for interpretation. In our conceptual framework for urban health, the urban context is defined by these socio-physical environments that ultimately influence urban health across all scale levels [18-20]. We focused exclusively on slum dwellers and considered three different levels: the neighbourhood, the household, and the individual level. We asked respondents: How would they generally rate their health (self-rated health)? And whether respondents had suffered from any disease within three months preceding the survey? We used the WHO-5 Well-being Index as a localised measure for self-rated mental well-being. The WHO-5 is a quick, reliable, and valid measure for assessing psychological well-being [21-25]. Although other indexes exist that are covering a wider spectrum of mental health status including mental illness such as the Beck Depression Inventory (BDI), comprised of 21 questions [26], the Centre for Epidemiological Studies Depression Scale (CES-D) [27] with 20 items, the General Health Questionnaire, composed of 12 questions (GHQ-12), or the Patient Health Questionnaire, composed of 9 questions (PHQ-9) [28], the WHO-5 is brief enough for population based studies [29]. The WHO-5 can thus easily be extended to a larger sample of slum population. It primarily targets hedonic well-being [29] by reflecting happiness rather than just the absence of depressive symptoms [25]. It was further successfully applied in both, developed [28,30-32] and developing countries [21,33,34]. Although the WHO-5 was not yet validated in Bangladesh, it was found reliable and effective among elderly Indian communities [21], which are socio-economically similar to Bangladeshi communities.

We hypothesised that the mental well-being of slum dwellers is associated with the social and physical environment even when controlling for the impact of personal factors such as age, gender and diseases.

## Methods

### Study design

We conducted a one-year cohort study in 2009 in nine slums of Dhaka. We analysed solely the baseline data (one point in time) of 1,938 adults (male = 48% and female = 52%) aged 15 to 99 years after excluding some respondents < 15 years of age. Face-to-face interviews by trained university graduates were performed.

An ethics waiver was granted from the ethics committee of the responsible medical association (Ärztekammer Westfalen-Lippe). According to their code of medical ethics at §15, paragraph 1, no further discussion with an ethics committee was needed due to the study's purely epidemiological character. However, we followed the guidelines and recommendations to assure Good Epidemiological Practice (GEP) as defined by the German Society for Epidemiology (DGEpi) [35]. Our cohort study was therefore conducted in accordance with ethical principles and respected human dignity as well as human rights. We did not use any medical equipment, collect any blood, or provided any placebo medicine. Our study strived to adequately involve the affected population groups and to report a qualified risk-communication to the interested public. We extensively discussed the aims and objectives of the survey with local community leaders first before inviting the residents to participate. We also discussed the aims of the survey with potential interviewees and proceeded only when their verbal consent was given. As such, everybody participated voluntarily in our survey and interviewees were free to answer. All information (e.g. health status) was self-rated or evaluated by the respondent. The interviews were conducted in the residents' private dwellings. However, we could not always guarantee that no neighbours were present at the time the interview was conducted. We planned our study with specific and concise research questions. The selection of the study population followed the main research question i.e., that the mental health of the slum residents is related to one's socio-physical environment and personal characteristics.

All efforts were made possible to accompany quality assurance of all relevant instruments and procedures. A detailed concept was developed in advance for the compilation and management of all data collected during the study, including the editing, plausibility verification, and coding of data. The analysis was carried out immediately after the survey using adequate methods. Methods, data, and results of our study were discussed in the context of existing evidence. Compliance with data protection regulations was respected in order to protect the right to informational self-determination. We used global positioning systems (GPS) to record the location of each interviewed household. This information was stored separately to the survey data and was only used anonymously in further analysis. Legal binding agreements were sought between all collaborators i.e., research colleagues and student collaborators from Jahangirnagar University, Centre for Urban Studies (CUS), Dhaka University (DU), Bangladesh University for Environment and Technology (BUET) each in Dhaka, Bangladesh, and Bielefeld University as well as Humboldt-Universität zu Berlin, both Germany.

### Sampling strategy for slums and households

Approximately 4,900 slum settlements in Dhaka were identified by the Centre for Urban Studies (CUS) in 2005 [11]. We used two criteria: minimum threshold value of 500 households and six acres of land per slum to select comparable slum settlements from the CUS survey. To achieve an adequate geographical distribution of the slum settlements, we subsequently selected administrative units that usually were not close to each other. In units with more than one slum, we randomly selected one of these settlements. We also adapted our selection process to account for evicted slums, or slums converted into affluent residential areas or open spaces since the CUS survey in 2005. A detailed map on Dhaka City, the cohort study and corresponding slum settlements can be found in Gruebner et al. [36].

### Sampling strategy for participants

To calculate the minimum sample size needed to gain a representative sample of families for each slum, we used a statistical formula (not given here) proposed by Bartlett et al. [37]. In our study, we used a 95% confidence level (i.e. alpha = 0.05) and an acceptable error margin of d = 6%. Since it was not possible to conduct a pilot study for estimating prevalences of our outcomes (p), we choose the recommended value of p = 0.50, which can provide maximum variance and maximum sample size. Our samples varied from slum to slum depending on the number of households in slums. To calculate the sampling rate r, we divided the number of families in the slum by the sample size. We then interviewed every rth household. When it was not possible to identify an interview partner at a household, we proceeded to the subsequent one and thereby achieved the target sample size.

### Explanatory variables

Baseline data from the cohort study were used (i.e. in a cross-sectional style). We structured the variables in the bio-geo-physical and the human-social environment. We conceptualised the variables at three different levels: the neighbourhood, the household, and the individual level, although they were measured and analysed at the individual level (cf. Tables 1, 2, 3 for details). We further generated geo-epidemiological variables through geoprocessing, conceptualised at the neighbourhood level but measured for each respondent/household separately (cf. Table 1).

**Table 1:** Physical neighbourhood characteristics

Characteristic	Mean	SD	Min	Max	SE	N
Distance to nearest river (in metre)	627.6	659.4	4.6	2448.3	15.1	1905
Distance to nearest street (in metre)	365.1	324.4	0.2	1225	7.4	1905
Distance to nearest park (in metre)	2477.2	2398.4	561.3	7910.2	54.95	1905
Vegetation ratio	5247.9	4374.7	118.1	22669.2	100.23	1905
Surface water ratio	807.54	1892.64	0	11750.9	43.4	1905
				**Category**	**%**	**N**
Is your area flood affected				Yes	69.2	1296
				No	30.8	576
Does your area have a proper drainage system				Yes	18.6	354
				No	81.4	1546

Descriptive statistics for variables used in the study. The metrical variables were gained through geoprocessing in GIS (geo-epidemiological variables), the categorical variables were gained through a cohort study in 2009 (baseline data).

**Table 2:** Socio-economic household characteristics

Characteristic	Mean	SD	Min	Max	SE	N
Monthly rent for the house (in Taka)	801.4	770.9	0	14000	17.7	1903
How many rooms do you have	1.89	2.2	0	19	0.1	1888
Family size (number of family members)	4.3	1.6	1	13	0.04	1905
Persons living in the same room	4	2.4	1	47	0.1	1905
Persons sharing same meals	4.4	2.5	1	54	0.1	1905
Family members earning income	1.7	0.9	0	6	0.02	1902
Monthly family income (in Taka)	6979.7	5149.5	0	140000	118	1905
Working hours per day	7.8	3.2	0	24	0.1	1894
How many family members smoke	0.7	0.6	0	6	0.01	1905
				**Category**	**%**	**N**
Light sufficiency in the house				Yes	26	495
				No	74	1408
Family has household item				Radio	5.7	109
				TV	33.4	635
				Gas burner	28.3	539
				Electric fan	69.4	1321
				Tape/CD/VCD	17.2	327
				Refrigerator	1.5	28
Is your house provisional or permanent				Provisional	91.5	1734
				Permanent	8.5	161
Room is used also for other purposes except living				Yes	30.1	568
				No	69.9	1317
Room is sufficient for family				Yes	23.2	433
				No	76.8	1435
Housing index				Kutcha	16.6	309
				Semi-pucca	52.9	986
				Pucca	30.5	567
Cooking material				Straw, wood	61.7	1174
				Kerosene	1.6	30
				Gas, electric	36.4	699
Type of water supply				Surface water	9.6	180
				Piped water	53.4	1004
Type of toilet facility				Open latrine	26.2	499
				Pit latrine	59.6	1135
				Septic tank	14.2	269
Type of garbage disposal				Open space	79.1	1501
				Bin outside house	13.5	256
				Collected	7.4	141
Do you have a job contract				Yes	4.8	91
				No	95.2	1798
Do you think that your job is harmful to your health				Yes	22.2	420
				No	77.8	1476
Do you like your job				I like it very much	5.5	103
				I like it	62.3	1169
				Its ok	18.1	340
				I don't like it	13	243
				I very much dislike it	1.1	21

Descriptive statistics for variables used in the study. The variables were gained through a cohort study in 2009 (baseline data).

**Table 3:** Health knowledge and behaviour

Characteristic	Category	N	%
Do you think that smoking tobacco is bad for your health	Yes	1855	97.8
	No	42	2.2
...physical exercise is good for your health	Yes	1833	97.2
	No	52	2.8
...polluted/clogged water/garbage near the house spread disease and increase the risk of poor health	Yes	1657	88.8
	No	209	11.2
...air pollution is bad for your health	Yes	1756	93.8
	No	116	6.2
Do you smoke cigarettes	Yes	475	24.9
	No	1430	75.1
Do you smoke inside your room	Yes	334	17.5
	No	1571	82.5
Community membership	Yes	170	9
	No	1726	91
Do you use a bed net	Yes	1873	98.4
	No	30	1.6
Education	0 years spent in school	1234	64.7
	1-5 years primary school	368	19.3
	6-10 years secondary school	180	9.5
	11+ years higher education	123	6.5
Marital status	Married	1700	89.2
	Not married/divorced/other	205	10.8
Migrant	Yes	1711	89.9
	No	193	10.1
Age group	15-24 years	419	22
	25-34 years	628	33
	35-44 years	430	22.6
	45-54 years	230	12.1
	55-64 years	131	6.9
	65-74 years	49	2.6
	75+ years	18	1
Gender	Female	983	51.6
	Male	922	48.4
Having had a disease	Yes	1469	77.4
	No	429	22.6

Descriptive statistics for variables used in the study. The variables were gained through a cohort study in 2009 (baseline data).

### Geoprocessing

**Figure 1:**
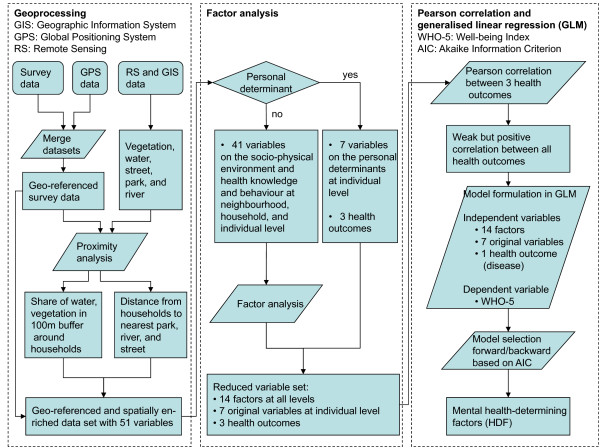
**Geo-epidemiological approach used for this study**. Parallelograms stand for geoprocessing or statistical processes, rhombuses for selection criteria and rectangles for outcomes. Note that levels were used only for conceptualising the socio-physical environment. All variables were available on the individual level, i.e. for each respondent separately and no aggregation to higher levels was done in order to prevent information loss.

We used data from Quickbird satellite imagery from January 22^nd ^2006 to estimate land cover properties. Based on this very high-resolution satellite data we calculated vegetation and water coverage in 100 m buffers around GPS-located households from the cohort study. In addition, distances from these household coordinates to the nearest river, street and park were calculated in GIS (Geographic Information System). Figure 1 presents a flowchart of our geo-epidemiological approach. All geoprocessing steps were done in ArcGIS version 9.3.1 [38].

### Health outcomes

We used five forced-choice Likert-scaled [39] questions (from zero to five) to derive the WHO-5. The five questions were summed up to an index ranging from 0 to 25 [40]. The WHO-5 is commonly used to assess psychological well-being and self-rated quality of life [25]. Commonly, WHO-5 values below 13 indicate poor mental well-being [40]. Additionally, we asked respondents how they rate their health with the possible aggregated answers "poor" and "fair (1)", "so-so" (2), and "good" as well as "excellent" (3). We subsequently term this variable the "self-rated health or SRH". As a third measure for health status we asked whether respondents had suffered from any disease in the three months preceding the survey, coded as 1 for yes and 0 for no, and shortly termed as "disease".

### Data analysis

Given the high number of potentially influential predictors we had to rely on statistical approaches to test the large number of hypotheses. Since the analysis of the pairwise correlation coefficients of our predictors indicated collinearity we had to tackle that issue. Therefore, we used factor analysis (FA) in SPSS (Version 17) to reduce our variable set to a fewer number of uncorrelated linear combinations of variables that contain most of the variance [41]. The factors were based on the correlation matrix. We extracted principal components and used a Varimax rotated solution with Kaiser Normalisation based on Eigenvalues greater than one [41,42]. The variables on age, gender, education, marital status, migration background, using bed net, and community membership were conceptualised as personal determinants being directly used in the multivariable generalised linear regression model. Hence, they were not included in FA. Health outcomes were also not included in the FA.

Associations between the set of independent variables (subsequently termed mental health-determining factors or HDF) and mental well-being (WHO-5) were studied using generalised linear regression models with a negative binomial distribution.

**Figure 2:**
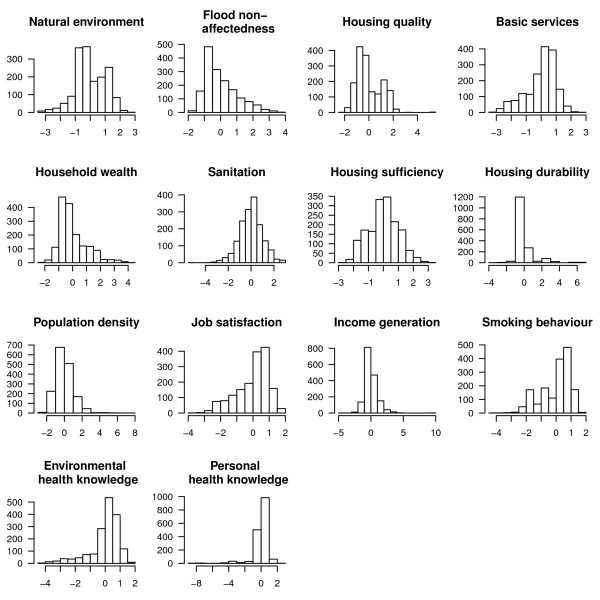
**Histograms for derived factors**. Descriptive statistics for the factors extracted through factor analysis in SPSS 17.

We included all HDF found through factor analysis and additionally the original variables for the individual level (for details refer to Tables 1, 2, 3 and Figure 2). We used the stepwise "stepAIC" algorithm available in the packet MASS in the statistical programming environment R [43] with both backward and forward selection using the Akaike Information Criterion [44] for model selection. We additionally calculated the intra-class correlation coefficient (ICC) in order to test for a hierarchical structure within the data. The ICC was 0.008, meaning that only 0.8% of the variance could be explained by the slum settlements alone. Hence we proceeded with a single level model instead of a multi level model.

## Results

### Geo-epidemiological variables

We generated five geo-epidemiological variables through geoprocessing in GIS, which we subsequently used in the factor analysis. See Table 1 for descriptive statistics on geo-epidemiological variables. Euclidean distances to the nearest major river, street and designated park areas were measured. Rivers were found to be within walking distance (1 km) for more than three-quarters of the households, and the nearest major street was also found to be within walking distance for 90% of the slum dwellers. Only the urban park areas were found to be beyond walking distances for the majority (90%) of the investigated households. We also found that the area within 100 metres around the houses of about 60% of the slum dwellers contained more than 10% green vegetation patches. In addition, 90% of the households were in neighbourhoods with less than 10% surface water, including rivers, lakes, and ponds.

We identified 14 factors, which we used as covariates in the generalised linear regression analysis. The factors represent the socio-physical environment and individual health knowledge and behaviour. The identified HDF explained 59.5% of the variance in the data, ranging from 6.3% (housing quality) to 3.4% (personal health knowledge) (cf. Table 4).

**Table 4:** Explanatory variables used for this study

Level		Health-determining factor (explained variance)	Original variables (Pearson correlation coefficients)
**Neighbourhood Physical environment**		Natural environment (4.3%)	○ Larger amounts of vegetation in 100 m around the households (0.8)
			○ Longer distances to the nearest major street (0.7)
			○ Lesser amounts of surface water in 100 m around the households (-0.6)
		
		Flood non- affectedness (4.1%)	○ Whether the area was regarded as flood non-affected (0.7)
			○ Whether the area was regarded as having a proper drainage system (0.7)
			○ Longer distances to the nearest river (0.5)

**Household**	**Physical environment**	Housing quality (6.3%)	○ Better-quality fuel for cooking (0.9)
			○ Owning a gas burner (0.8)
			○ Higher monthly rent for the house (0.6)
			○ Better construction materials (0.5)
		
		Access to basic services (4.7%)	○ Owning an electric fan (0.6)
			○ Short distance to the nearest river (0.5)
			○ Better water supply (0.5)
			○ Large distance to the nearest park area
			(-0.8)
		
		Sanitation (3.6%)	○ Better toilet facility (0.7)
			○ Better garbage disposal (0.6)
		
		Housing sufficiency (3.6%)	○ Whether the room was used for other purposes aside from living (0.7)
			○ Sufficient light in the house (0.6)
			○ Whether the room was regarded as sufficient for one's family (0.5)
		
		Housing durability (3.5%)	○ Whether the house was considered to be permanent (0.8)
			○ Household had a refrigerator (0.7)
	
	**Economic environment**	Household wealth (4.3%)	○ Owning a Tape/CD/VCD (0.7)
			○ Owning a radio (0.6)
			○ Owning a TV (0.6)
			○ Higher number of rooms (0.5)
		
		Job satisfaction (4%)	○ Not thinking that the job is harmful to one's health (0.8)
			○ Liking one's job (0.7)
			○ Fewer working hours per day (-0.4)
		
		Income generation (3.7%)	○ A large number of family members earning income (0.7)
			○ Having a job contract (0.4)
			○ Higher monthly family income (0.7)
			○ Working more hours a day (0.2)
	
	**Social environment**	Population density (5.2%)	○ Higher number of family members (0.8)
			○ Higher numbers of persons sharing the same meals (0.7)
			○ Higher number of persons living in the same room (0.7)

**Individual**		Smoking behavior (4.8%)	○ Not smoking cigarettes (0.8)
			○ Not smoking inside the room (0.8)
			○ Small number of family members who smoke (-0.7)
		
		Environmental health knowledge (3.9%)	○ Thinking that polluted, stagnant water and garbage near one's house could spread disease and increase the risk of poor health (0.8)
			○ And that air pollution is bad for one's health(0.6)
		
		Personal health knowledge (3.4%)	○ Thinking that smoking tobacco is bad for one's health (0.7)
			○ And that physical exercise can be good for one's health (0.7)
		
		Community	Original variables were used
		member	
		Using bed net	
		Education	
		Married	
		Migrant	
		Age	
		Gender	
		Disease	

Mental health-determining factors (HDF) were used as explanatory variables. They were prior extracted through factor analysis in SPSS 17. In the table, we report the name of each HDF (i.e. the factors) and in brackets the explained variance. The original variables which were found to be correlated with these HDF are also displayed, with Pearson correlation coefficients in brackets.

### Health outcomes

**Figure 3:**
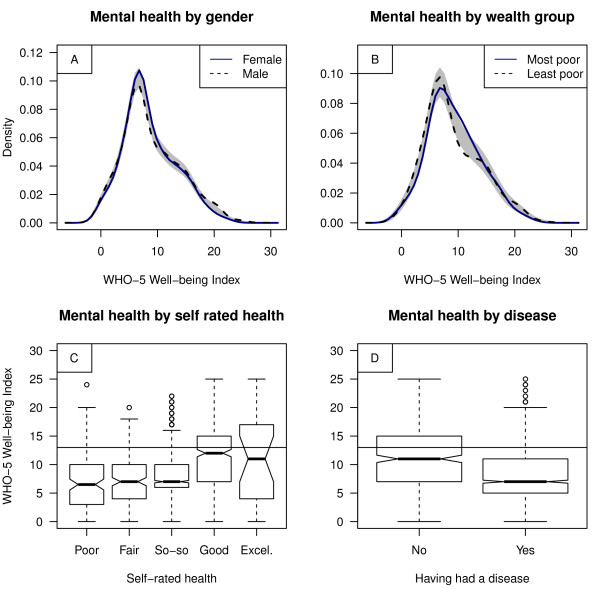
**Descriptive statistics for WHO-5 scores (mental well-being), self-rated health and diseases**. For A and B, a bootstrap hypothesis test of equality between the both groups was applied with gender being equal (p value = 0.55) and wealth group being significantly different from each other (p value = 0.04), indicated by a reference band in grey. For B, least poor implies to the upper wealth index quintile, while most poor implies to the lowest. Note that for C and D, a WHO-5 scored 13 or above has been found to be indicative of good mental well-being in high-income country settings (cf. horizontal line).

Good mental well-being was found in 20% of the total population sample (n = 1,644) i.e., a WHO-5 scored 13 or above, which has been found to be indicative of mental well-being in high-income country settings. These scores were found in 21% for females and 25% for males and 25% for the most poor (lower household wealth quintile) and 26% for the least poor (upper household wealth quintile) population group (cf. Figure 3A, B). Slum dwellers rated their health mainly as "so-so" (56%), whereas good or excellent health status was reported by 26% of the females, 26% of the males, 27% in the most poor, and 30% in the least poor population group. We found that WHO-5 scores were positively correlated with self-rated health (Pearson correlation coefficient = 0.32, p < 0.001). Similarly, 78% of the females and 76% of the males reported that they had had a disease in the three months preceding the survey. WHO-5 scores were hence negatively correlated with 'having had a disease in the three months preceding the survey' (Pearson correlation coefficient = -0.24, p < 0.001) (cf. Figure 3C, D).

### Mental well-being

We identified several HDF from the socio-physical environment as well as health knowledge and behaviour having significant associations with mental well-being (Table 5). Furthermore, personal determinants like gender, age, and disease were significantly associated with mental well-being. We found the strongest positive association with environmental health knowledge and male gender. Furthermore, mental well-being was positively associated with lower flood risk (flood non-affectedness), higher quality, sufficiency, and durability of the house, better sanitation and income generation ability, as well as with job satisfaction. A strong negative association was found for respondents who had suffered from any disease in the three months prior to the survey. Mental well-being was further found to be negatively associated with age, better personal health knowledge, higher population density, and selected features of the natural environment.

**Table 5:** Determinants of mental well-being (WHO-5)

Level		Mental health- determining factor (HDF)	Multivariable generalized linear regression	
			Coefficient	95%CI LL/UL
**Neighbourhood Physical environment**		Natural environment	-0.06***	-0.08/-0.03
		Flood non-affectedness	0.06***	0.04/0.09

**Household**	**Physical environment**	Housing quality	0.03*	0.01/0.06
		Basic services	---	---
		Sanitation	0.08***	0.06/0.11
		Housing sufficiency	0.07***	0.04/0.09
		Housing durability	0.07***	0.05/0.09
	
	**Economic Environment**	Household wealth	---	---
		Job satisfaction	0.09***	0.06/0.11
		Income generation	0.08***	0.06/0.11
	
	**Social Environment**	Population density	-0.05***	-0.07/-0.02

**Individual**		Smoking behaviour	---	---
		Environmental health	0.11***	0.08/0.13
		knowledge		
		Personal health	-0.03*	-0.05/-0.004
		knowledge		
		Community member	0.07.	-0.02/0.15
		Using bed net	---	---
		Education	---	---
		Married	---	---
		Migrant	0.06.	-0.02/0.15
		Age	-0.01***	-0.01/-0.004
		Gender:		
		Female	Reference	
		Male	0.11***	0.06/0.16
		Having had a disease:		
		No	Reference	
		Yes	-0.22***	-0.28/-0.16
Significance codes: < 0.001 '***',< 0.01 „**',< 0.05 '*',> 0.05 '.', not in model '---' CI: Confidence interval with, LL=Lower limit and, UL=Upper limit				

A multivariable generalised linear regression model was used assuming a negative-binomial distribution of the target variable mental well-being (WHO-5 score). The explaining variables (HDF) are displayed in the table. A forward/backward model selection approach was used based on AIC. Note that despite conceptualising the HDF at different levels, all analyses were done at the individual level.

We could not find any associations between mental well-being and basic services, household wealth, smoking behaviour, actively participating within the community, using bed net, the level of education, marital status, and migration background.

## Discussion

The socio-physical environments of slums are diverse and can compromise and support health in a variety of ways. We analysed the determinants of mental well-being among slum dwellers in Dhaka. We found that mental well-being was unequally distributed among the population and younger, male, and more affluent dwellers enjoyed better health [cf. [10,45,46]]. Furthermore, physical well-being was associated with mental well-being [cf. [3]]. This study adds evidence regarding factors determining mental well-being of slum residents in Dhaka and hence in comparable settings worldwide.

At the individual level mental well-being was positively associated with environmental health knowledge, which reflects a person's awareness of environmental threats (i.e., that polluted, stagnant water and garbage near one's house could spread disease and that air pollution increases the risk of poor health). Such knowledge may justify protective measures and eventual adaptation strategies of the local residents. An interesting fact is the observed negative relationship between mental well-being and personal health knowledge, which reflects a person's awareness of the effects of personal sedentary lifestyles and other activities that can cause poor health, such as smoking or less physical exercise. One explanation for this relationship could be that a higher awareness of health issues might cause a tendency to be dissatisfied with the overall poor living conditions.

Actively participating within the community may play a role when the focus is on eudaemonic well-being, which is a concept incorporating for example positive relation with others [29,47]. Weich et al. [29] for example, found in a sample of adults in England that items like getting on with the family or belonging and enjoying spare time were more related to eudaemonic well-being. As a hedonic concept of mental well-being however, the WHO-5 focuses on happiness, which was in the case of Dhaka not related to actively participating in the local community. Further individual level factors such as smoking behaviour, using bed net, the level of education, marital status, and migration background did also not contribute to the slum dwellers' mental well-being in Dhaka.

Although having measured all variables at the individual level, we conceptualised the HDF at the different levels for ease of interpretation. Most HDF at the household level, for instance, relate to the built environment. Unfavourable housing quality is thereby assumed to cause poor health by provoking asthma and other respiratory conditions, injuries, psychological distress, or by hindering child development [48]. Good sanitation (i.e., garbage disposal and the quality of the toilet facility) can decrease the risk of infectious disease and other ailments, such as gastro intestinal diseases or respiratory diseases [48]. In accordance with these relationships, mental well-being in the slums of Dhaka was positively associated with good sanitation. Furthermore, the quality, sufficiency, and durability of housing were found to be positively associated with mental well-being. In contrast, household's wealth reflected by the household's number of available rooms and items such as radio or TV plaid no role in shaping the mental well-being of slum residents in Dhaka. Neither did the households' availability of basic services, such as water and electricity supply. However, each of the predictors from the built environment could capture the socio-economic status (SES) of an individual or household that is well known to be associated with mental well-being [45,49,50]. These predictors can define the frame of action within which a household can respond to health threats [19,51]. Hence, these factors could also be conceptualised as belonging to the economic environment. In any case, these factors may shape the intrinsic ability of an individual or household to resist or cope with the impact of a possible physical or social event [51] and were therefore crucial determinants of mental well-being in our study. Furthermore, our study revealed a positive association of well-being with income generation and job satisfaction, describing the ability to generate income as well as satisfaction and safety at work. More than 80% of adult slum dwellers are engaged in the informal sector which provides a means of survival for a substantial section of the workforce [52]. This sector offers a flexible labour market, absorbs most of the workforce and provides income-generating opportunities and services for a large number of unskilled and manual labours [53]. Although the informal sector substantially contributes to the national and urban economy [54,55], informal sectors are often associated with unfavourable conditions with regard to e.g. working and living conditions, pollutants, discrimination, exploitation, income, occupational safety, and legal and social security [19,56]. Against this background, it becomes clear why good income generation and job satisfaction showed up as important predictors for good mental well-being among Dhaka's slum dwellers.

For mental well-being, population density was also an important factor in our study. We hypothesised that in the slums of Dhaka, crowding put enormous stress on residents with consequent implications for mental well-being, possibly due to a lack of privacy. Other studies showed that social norms in densely populated urban areas may further support individual or group behaviours that affect health outcomes (e.g. smoking, diet, exercise, sexual behaviour) [20].

For a profound discussion on the association between mental well-being and the 'natural environment' as well as with flood non-affectedness, refer to Gruebner et al. [36]. In brief, the risk of flooding is relatively common in Dhaka due to its unique low lying geography and its extremely rapid urban growth combined with an urban mismanagement [36,57-59]. In the slums, vegetation cover is scarce, and green areas turned out to be low-lying and regularly flooded areas. Combined with poor sanitation, open waste water drainage and garbage disposal, such vegetation patches increase the risk for infectious diseases (e.g., diarrhoea). Our analysis thus identified environmental disservices [60] rather than services, which shall explain the negative association of the HDF natural environment and mental well-being as well as the positive association with flood non-affectedness.

## Limitations

We included a large number of variables from different sources and at different levels. However, the natural environmental variables drawn from satellite analysis preceded the survey-based outcome variables by three years. But both data sources were collected in the same season in order to obtain similar phenological and hydrological situations and thus the temporal mismatch is unlikely to cause large biases in the results. Second, given the large number of potential influential predictors and the limited knowledge on HDF in the slums of Dhaka we had to test a large number of hypotheses using a stepwise model selection procedure. This method has been criticized [61] and the share of concerns raised. Nevertheless, we had to rely on the method since there were no obvious testable sets of predictors. The results should be interpreted with respect to that: the selected regression model can be considered the best fitting model but other models with slightly worse selection criteria exist. Third, the nine selected slums may not fully represent the ~4900 slums of Dhaka. Fourth, the replacement of respondents (interviewees) for the selected households (based on our systematic sampling) by the respondents from the subsequent houses was done to achieve our sample target. This sampling procedure may cause bias, yet, the share of non-respondents was very small and thus this bias is negligible. Fifth, although the WHO-5 is a reliable measure for psychological well-being [25], it has thus far not been validated in a slum of a developing country. More effort should be invested by specialists in this research domain in order to provide reliability studies from the psychological point of view. Sixth, we might have missed some influential HDF in our model (e.g., air pollution, social capital, or accessibility to health care facilities). Seventh, the cross-sectional and quantitative nature of our study could certainly not cover all aspects of mental well-being. However, our study revealed plausible results which could be embedded with other studies on health and the environment. The study of mental well-being in slums should be further supported by longitudinal quantitative studies in combination with qualitative research. Furthermore, multilevel studies are needed to assess the neighbourhood effects of e.g. SES in slums, which are likely to affect health status independently from personal SES as has been shown by many studies in higher income countries' cities [62].

## Conclusions

From this study we could inform about the status of mental well-being in Dhaka's slums. The important factors that determine the mental well-being relate to the socio-economic (job satisfaction, income generation ability, population density) and physical environment (environmental pollution, lower flood risk, better sanitation and quality, sufficiency and durability of the house). Individual level characteristics such as diseases, gender, and knowledge upon environmental health threats are important mental well-being determinants. Given that mental well-being is associated with physical well-being, our study provides crucial information for developing better health care and disease prevention programmes in slums of Dhaka and comparable settings worldwide.

## Competing interests

The authors declare that they have no competing interests.

## Authors' contributions

OG carried out the cohort study, designed the paper, performed statistical analyses and drafted the manuscript. MMHK designed and carried out the cohort study and helped in interpreting the findings. SL and DM participated in the design of the paper and guided the statistical analysis and interpretation. AK and PH obtained the grant, designed the overall framework and coordination, and helped to draft the manuscript. TL helped in designing the manuscript and revised it critically. All authors read and approved the final manuscript.

## Pre-publication history

The pre-publication history for this paper can be accessed here:

http://www.biomedcentral.com/1471-2458/12/177/prepub
